# Supplementary material for: Asymptomatic carriage of intestinal protists is common in children in Lusaka Province, Zambia
Source: PLoS Negl Trop Dis. 2024 Dec 13;18(12):e0012717. doi: 10.1371/journal.pntd.0012717 (PMC11676895; doi:10.1371/journal.pntd.0012717)
Supplement: S1 Table — (DOCX) [file pntd.0012717.s001.docx]

**Supplementary Table 1.** Oligonucleotides used for the molecular identification and/or characterization of the intestinal protists investigated in the present study.

| **Target organism** | **Locus** | **Oligonucleotide** | **Sequence (5´–3´)** | **Reference** |
| --- | --- | --- | --- | --- |
| *Giardia duodenalis* | *ssu* rRNA | Probe | FAM–CCCGCGGCGGTCCCTGCTAG–BHQ1 | [44] |
|  |  | Gd-80F | GACGGCTCAGGACAACGGTT |  |
|  |  | Gd-127R | TTGCCAGCGGTGTCCG |  |
|  | *ssu* rRNA | Gia2029 | AAGTGTGGTGCAGACGGACTC | [45] |
|  |  | Gia2150c | CTGCTGCCGTCCTTGGATGT |  |
|  |  | RH11 | CATCCGGTCGATCCTGCC | [46] |
|  |  | RH4 | AGTCGAACCCTGATTCTCCGCCAGG |  |
|  | *gdh* | GDHeF | TCAACGTYAAYCGYGGYTTCCGT | [47] |
|  |  | GDHiF | CAGTACACCTCYGCTCTCGG |  |
|  |  | GDHiR | GTTRTCCTTGCACATCTCC |  |
|  | *bg* | G7_F | AAGCCCGACGACCTCACCCGCAGTGC | [48] |
|  |  | G759_R | GAGGCCGCCCTGGATCTTCGAGACGAC |  |
|  |  | G99_F | GAACGAACGAGATCGAGGTCCG | [49] |
|  |  | G609_R | CTCGACGAGCTTCGTGTT |  |
|  | *tpi* | AL3543 | AAATIATGCCTGCTCGTCG | [50] |
|  |  | AL3546 | CAAACCTTITCCGCAAACC |  |
|  |  | AL3544 | CCCTTCATCGGIGGTAACTT |  |
|  |  | AL3545 | GTGGCCACCACICCCGTGCC |  |
| *Cryptosporidium* spp. | *ssu* rRNA | CR-P1 | CAGGGAGGTAGTGACAAGAA | [51] |
|  |  | CR-P2 | TCAGCCTTGCGACCATACTC |  |
|  |  | CR-P3 | ATTGGAGGGCAAGTCTGGTG |  |
|  |  | CPB-DIAGR | TAAGGTGCTGAAGGAGTAAGG |  |
|  | *gp60* | AL-3531 | ATAGTCTCCGCTGTATTC | [52] |
|  |  | AL-3535 | GGAAGGAACGATGTATCT |  |
|  |  | AL-3532 | TCCGCTGTATTCTCAGCC |  |
|  |  | AL-3534 | GCAGAGGAACCAGCATC |  |
| *Entamoeba histolytica* | *ssu* rRNA | Probe | FAM–TCATTGAATGAATTGGCCATTT–MGB | [54] |
| *Entamoeba dispar* |  | Probe | VIC–TTACTTACATAAATTGGCCACTTTG–MGB |  |
| *Entamoeba histolytica*/*dispar* |  | Ehd-239F | ATTGTCGTGGCATCCTAACTCA |  |
|  |  | Ehd-88R | GCGGACGGCTCATTATAACA |  |
| *Blastocystis* sp. | *ssu* rRNA | BhRDr | GAGCTTTTTAACTGCAACAACG | [55] |
|  |  | RD5 | ATCTGGTTGATCCTGCCAGT |  |
|  | *ssu* rRNA | ILMN_Blast505_532F | TCGTCGGCAGCGTCAGATGTGTATAAGAGACAGGGAGGTAGTGACAATAAATC^a^ | [56] |
|  |  | ILMN_Blast998_1017R | GTCTCGTGGGCTCGGAGATGTGTATAAGAGACAGTGCTTTCGCACTTGTTCATC**^a^** |  |

^a^Primer used in next-generation amplicon sequencing. Adapter sequences are shown underlined.

*bg*: β-giardin; *gdh*: Glutamate dehydrogenase; *gp60*: 60 kDa glycoprotein; ITS: Internal transcribed spacer; *ssu* rRNA: Small subunit ribosomal RNA; *tpi*: Triose phosphate isomerase.
