# Supplementary material for: Asymptomatic carriage of intestinal protists is common in children in Lusaka Province, Zambia
Source: PLoS Negl Trop Dis. 2024 Dec 13;18(12):e0012717. doi: 10.1371/journal.pntd.0012717 (PMC11676895; doi:10.1371/journal.pntd.0012717)
Supplement: S2 Table — (DOCX) [file pntd.0012717.s002.docx]

**Supplementary Table 2.** PCR cycling conditions used for the molecular identification and/or characterization of the intestinal protists investigated in the present study.

|  |  | **Temperature and time** | | | |  |  |  |
| --- | --- | --- | --- | --- | --- | --- | --- | --- |
| **Target organism** | **Locus** | **Initial denaturation** | **Denaturation** | **Annealing** | **Extension** | **No. cycles** | **Final extension** | **Reference** |
| *Giardia duodenalis* | *ssu* rRNA | 95°C 15 min | 95°C 15 s | 60°C 1 min | 72°C 30 s | 45 | – | [44] |
|  | *ssu* rRNA | 95°C 2 min | 95°C 45 s | 58/55°C 30 s | 72°C 45 s | 35 | 72°C 4 min | [45] |
|  | *gdh* | 95°C 3 min | 95°C 30 s | 55°C 30 s | 72°C 1 min | 35 | 72°C 7 min | [47] |
|  | *bg* | 95°C 7 min | 95°C 30 s | 65/55°C 30 s | 72°C 1 min | 35 | 72°C 7 min | [49] |
|  | *tpi* | 94°C 5 min | 94°C 45 s | 50°C 45 s | 72°C 1 min | 35 | 72°C 10 min | [50] |
| *Cryptosporidium* spp. | *ssu* rRNA | 94°C 3 min | 94°C 40 s | 50°C 40 s | 72°C 1 min | 35 | 72°C 10 min | [51] |
|  | *gp60* | 95°C 5 min | 94°C 45 s | 59/50°C 45 s | 72°C 1 min | 35 | 72°C 10 min | [52] |
| *Entamoeba histolytica* | *ssu* rRNA | 95°C 15 min | 95°C 15 s | 60°C 1 min | 72°C 30 s | 45 | – | [53] |
| *Blastocystis* sp. | *ssu* rRNA | 95°C 3 min | 94°C 1 min | 59°C 1 min | 72°C 1 min | 30 | 72°C 2 min | [55] |

*bg*: β-giardin; *gdh*: Glutamate dehydrogenase; ITS: Internal transcribed spacer; *gp60*: 60 kDa glycoprotein; *ssu* rRNA: Small subunit ribosomal RNA; *tpi*: Triose phosphate isomerase.
