# Supplementary material for: Asymptomatic carriage of intestinal protists is common in children in Lusaka Province, Zambia
Source: PLoS Negl Trop Dis. 2024 Dec 13;18(12):e0012717. doi: 10.1371/journal.pntd.0012717 (PMC11676895; doi:10.1371/journal.pntd.0012717)
Supplement: S4 Table — Lusaka Province, Zambia (2023). GenBank accession numbers are provided. (DOCX) [file pntd.0012717.s004.docx]

**Table S4.** Frequency and molecular diversity of *G. duodenalis* identified at the *ssu* RNA, *gdh*, *bg*, and *tpi* loci in the paediatric population under study. Lusaka Province, Zambia (2023). GenBank accession numbers are provided

| **Marker** | **Assemblage** | **Sub-assemblage** | **No. isolates** | **Reference sequence** | **Stretch** | **Single nucleotide polymorphisms** | **GenBank ID** |
| --- | --- | --- | --- | --- | --- | --- | --- |
| *ssu* rRNA | A | – | 2 | M54878 | 1–292 | None | PQ185660 |
|  |  | – | 1 | M54878 | 1–292 | C102Y | PQ185661 |
|  |  | – | 1 | M54878 | 1–288 | G267S | PQ185662 |
|  | B | – | 16 | AF199447 | 1–293 | None | PQ185663 |
|  |  | – | 2 | AF199447 | 1–293 | G71R | PQ185664 |
|  |  | – | 1 | AF199447 | 1–293 | T193W | PQ185665 |
|  |  | – | 4 | AF199447 | 1–289 | G268C | PQ185666 |
|  |  | – | 2 | AF199447 | 1–291 | G268S | PQ185667 |
|  | A/B | – | 2 | – | – | – | – |
| *gdh* | A | AII | 5 | L40510 | 66–491 | None | PQ213645 |
|  |  | BIII | 1 | AF069059 | 40–389 | C99T, T147C, C309T | PQ213646 |
|  |  | BIV | 1 | L40508 | 76–491 | T96G, T183Y, T387C, C432Y, A438R | PQ213647 |
|  |  |  | 1 | L40508 | 76–491 | C123Y, T135Y, T183C, G186R, T387C, C432Y, A438R | PQ213648 |
|  |  |  | 1 | L40508 | 77–430 | T183C, C216T, T387C | PQ213649 |
|  |  |  | 2 | L40508 | 76–491 | T183C, T387C, C396T,C423T | PQ213650 |
|  |  |  | 1 | L40508 | 76–491 | T183Y, T387Y, C396Y,C423Y | PQ213651 |
|  |  |  | 1 | L40508 | 76–491 | T183Y, T387Y, C396Y,C423Y, C432Y, A438R | PQ213652 |
|  |  |  | 1 | L40508 | 76–494 | T183C, C411T | PQ213653 |
|  |  |  | 1 | L40508 | 76–491 | T387C, A438G | PQ213654 |
|  |  |  | 1 | L40508 | 76–491 | C423T | PQ213655 |
|  |  | BIII/BIV | 1 | L40508 | 76–491 | T135Y, T183Y, G186R, C255T, C273T, T312Y, C345Y, T366Y, C372Y, T387Y, G402R, A438G | PQ213656 |
|  |  |  | 2 | L40508 | 76–491 | T135Y, T183Y, C255Y, C273Y, C345Y, T366Y, T387C, C396Y, C423Y, A438R, T462Y | PQ213657 |
|  |  |  | 1 | L40508 | 76–491 | T183Y, G186R, C255Y, C273Y, G276R, C333Y, T366Y, T387C, C396Y, C423Y, A438R, T462Y | PQ213658 |
|  |  |  | 1 | L40508 | 134–485 | C255Y, C273Y, G377R, T387C, A438R | PQ213659 |
| *bg* | A | AIII | 4 | AY072724 | 102–604 | None | PQ213660 |
|  | B | – | 1 | AY072727 | 98–603 | C156Y, A183R, C309Y | PQ213661 |
|  |  | – | 1 | AY072727 | 102–603 | C165Y, A183R, G286R, C309Y, T519Y, C564Y | PQ213662 |
|  |  | – | 2 | AY072727 | 93–604 | C165Y, C309Y, G459R, C483Y | PQ213663 |
|  |  | – | 1 | AY072727 | 102–593 | C165Y, A228R, C309T | PQ213664 |
|  |  | – | 1 | AY072727 | 96–591 | A183G, A228G, T519C, C564T | PQ213665 |
|  |  | – | 1 | AY072727 | 93–596 | A232R | PQ213666 |
|  |  | – | 2 | AY072727 | 93–594 | C309T | PQ213667 |
|  |  | – | 3 | AY072727 | 98–753 | C309Y | PQ213668 |
|  |  | – | 1 | AY072727 | 97–590 | C450T | PQ213669 |
| *tpi* | A | AII | 2 | U57897 | 292–805 | None | PQ213670 |
|  |  |  | 2 | U57897 | 275–805 | C287G | PQ213671 |
|  | B | BIII | 1 | AF069561 | 1–456 | C34T, G54A, C108T, C141T, G189A | PQ213672 |
|  |  |  | 1 | AF069561 | 1–456 | C34Y, C43Y, G105R, C108Y, G189R, A223R, A376R | PQ213673 |
|  |  |  | 1 | AF069561 | 1–456 | C34Y, C43Y, G105R, C108Y, G189R, A376R | PQ213674 |
|  |  |  | 1 | AF069561 | 1–456 | G168R, A262G | PQ213675 |
|  |  |  | 1 | AF069561 | 1–436 | G390R | PQ213676 |
|  |  | BIII/BIV | 1 | AF069560 | 1–479 | A5R, T11Y, T134Y, C144Y, C164Y, A176R, G212R, C237Y, A246R, A263R, A368R, A395G, A437M, A449R, G476R | PQ213677 |
|  |  |  | 1 | AF069560 | 1–479 | A5R, T11Y, T134Y, C144Y, A176R, G212R, C237Y, A246R, A263R, A368R, A395G, A437M, A449R, G476R | PQ213678 |
|  |  |  | 1 | AF069560 | 1–479 | A5R, G17K,T29Y, T57Y, T86Y, T100Y, T131Y, T134Y, T141Y, C164Y, A176R, G235R, T266Y, T299Y, C349Y, A395G | PQ213679 |
|  |  |  | 1 | AF069560 | 1–479 | A5R, T57Y, G128R, T131Y, T134Y, A176R, G207R, G212R, A395G, A449R | PQ213680 |
|  |  |  | 1 | AF069560 | 1–479 | C38T, T57Y, C68Y, T131Y, T134Y, G268R, A395G | PQ213681 |

*bg*: β-giardin; *gdh*: Glutamate dehydrogenase; M: C/A; R: A/G; *ssu* rRNA: Small subunit ribosomal RNA; *tpi*: Triose phosphate isomerase; W: A/T; Y: C/T.
