# Supplementary material for: Asymptomatic carriage of intestinal protists is common in children in Lusaka Province, Zambia
Source: PLoS Negl Trop Dis. 2024 Dec 13;18(12):e0012717. doi: 10.1371/journal.pntd.0012717 (PMC11676895; doi:10.1371/journal.pntd.0012717)
Supplement: S5 Table — Lusaka Province, Zambia (2023). GenBank accession numbers are provided (DOCX) [file pntd.0012717.s005.docx]

**Table S5.** Frequency and molecular diversity of *Cryptosporidium* spp. identified at the *ssu* RNA locus in the paediatric population under study. Lusaka Province, Zambia (2023). GenBank accession numbers are provided.

| **Species** | **No. isolates** | **Reference sequence** | **Stretch** | **Single nucleotide polymorphisms** | **GenBank ID** |
| --- | --- | --- | --- | --- | --- |
| *Cryptosporidium hominis* | 2 | AF108865 | 575–900 | None | PQ191442 |
| *Cryptosporidium parvum* | 1 | AF112571 | 543–1030 | A646G, 647_649DelATT, T654C, T663C, 686_689DelTAAT | PQ191443 |
|  | 7 | AF112571 | 527–1029 | A646G, T649G, 686_689DelTAAT, T693A | PQ191444 |
|  | 1 | AF112571 | 524–956 | A646G, T649G, 686_689DelTAAT, T693A, A722G, A815G | PQ191445 |

*ssu* rRNA: Small subunit ribosomal RNA.
