# Supplementary material for: Asymptomatic carriage of intestinal protists is common in children in Lusaka Province, Zambia
Source: PLoS Negl Trop Dis. 2024 Dec 13;18(12):e0012717. doi: 10.1371/journal.pntd.0012717 (PMC11676895; doi:10.1371/journal.pntd.0012717)
Supplement: Attachment — Submitted filename: Review_PNTD-D-24-01392.pdf [file pntd.0012717.s006.pdf]

This study evaluated the asymptomatic intestinal protozoan infection in Zambia. High-accuracy molecular diagnostic approaches, including specific PCR and next-generation sequencing, were first utilized in this survey. The findings provided the prevalence of *Cryptosboridium* sp., *Giardia duodenalis*, *Entamoeba dispar*, and *Blastocystis* sp. in school-aged healthy individuals in Chongwe (158) and Kafue (109) districts in Lusaka Province. In addition, detailed information on subtypes and genetic variations was also documented. This investigation highlights the importance of asymptomatic carriers of intestinal protists in public health in the sub-Sahara area.

Generally, the manuscript seems almost acceptable in the current version; however, some issues should be addressed before further processing.

1. Lines 33 to 38:
   The samples were collected from Kafue and Chongwe districts in Lusaka Province, Zambia. The epidemiology investigation provides valuable information for African countries and regions with similar geographical characteristics. Therefore, a map containing the geographical, hydrological, and livestock-keeping facilities in Lusaka Province, especially the Kafue and Chongwe districts, is recommended to be provided.
2. Page 6 to lines 13:
   It is appreciated that the authors organized a list to emphasize the previous epidemiological surveys and the utilities of relatively low-sensitivity methods in Zambia (Table 1). However, the table, if possible, should be introduced/ discussed more or moved as a supplementary file.
3. Lines 169 to 173:
   The co-infection of individuals with two or more protists was mentioned. However, further analysis was expected, such as the origin of samples, the livestock contact, etc. The discussion section should also include the possible speculation of the co-infection with different protists.
4. Lines 187 to 191:
   The authors mentioned that “all 79 faecal DNA samples with a positive result …”, but only 31 could be amplified and typed. The detailed detection strategy was stated in the method section (from line 70). However, for clarity and to make the audience easy to understand, the screening process needed to be introduced in the result section, too.
5. Lines 244 to 249:
   The authors stated that “A total of 97 *Blastocystis*-positive samples were

successfully genotyped", and the proportion of subtypes was revealed. It was unknown why the following paragraph started with "a subset of samples (n=45) …". What were the missing samples out of the subset 45? Again, although the method section was stated, the origin of the subset should also be mentioned logically in the result section.

6. Lines 268 to 276:
   The whole paragraph about intra-subtype variants, coupled with Table 5, is not easy to understand.

   Table 5 showed the subtype, No. of unique ST variants, No. of samples containing variants, and GenBank accession number. Nonetheless, "… with 27 unique variants among 28 ST2-positive samples…", "… with 19 unique variants among the 23 ST1-positive samples", and "…with only six unique variants among 14 ST2-positive samples": these "positive samples (28, 23, and 14 positive samples mentioned above)" could not be found in the table.

   Moreover, "…relatively evenly distributed variants among samples", "with only three unique variants", and "… detected in more than five children" (lines 272 to 273): The statements "three unique variants" and "more than five children" were not found Table 5.

   Lines 274 to 276: "the multiple unique variants of ST1 and ST2…" and "multiple variants of ST3 were only detected in a single sample". The same issue as above, the sample information was absent.

   Most importantly, it was easy to imagine that NGS generated massive amounts of data, and the authors tried to explain all the valuable information in this manuscript. However, the authors did not mention the reason for discussing unique intra-subtype variants within samples.
7. Lines 317 to 323: the paragraph should be moved to line 365, where the *Blastocystis* was discussed.
8. The authors tried to elucidate sub-assemblages and subtypes of *G. duodenalis* and *Blastocystis* sp. as possible, respectively. Intriguingly, detection of the sub-assemblages of *G. duodenalis* at least 6 rounds of PCR were performed, whereas NGS was conducted to determine the subtypes of *Blastocystis* sp.. It was noticed that the NGS was solely designed for *Blastocystis* sp. identification; why did the authors not conduct NGS to detect both protists, especially since they caused the most frequent co-infection in tested samples?
9. The NGS-based subtype determination of *Blastocystis* sp. was performed in this study. It is known that the protist might not be pathogenic or even beneficial for human hosts. Was it worth the effort to determine the subtypes?
