# Supplementary material for: Asymptomatic carriage of intestinal protists is common in children in Lusaka Province, Zambia
Source: PLoS Negl Trop Dis. 2024 Dec 13;18(12):e0012717. doi: 10.1371/journal.pntd.0012717 (PMC11676895; doi:10.1371/journal.pntd.0012717)
Supplement: Attachment — Submitted filename: Rebuttal letter Manuscript PNTD-D-24-01392.docx [file pntd.0012717.s007.docx]

**Asymptomatic carriage of intestinal protists is common in children in Lusaka Province, Zambia**

PNTD-D-24-01392

Mutengo et al.

**Reviewer #1**

The manuscript presents data on a relevant topic and is an attempt to fill a gap in the literature for a topic that has largely been neglected in public health research, particularly in sub-Saharan Africa. Authors have used not only PCR and Sanger sequencing to perform the typical detection and basic genotyping of intestinal protists, but have also used a new approach (NGS amplicon sequencing) to explore and uncover mixed infections by *Blastocystis*. The manuscript is well written, however it can benefit from some minor adjustments and corrections, as suggested in the points that I raise below.

Reply: We thank Reviewer #1 for his/her initial positive appraisal of manuscript PNTD-D-24-01392. We have considered all the comments and suggestions raised during the appraisal of the manuscript and thoroughly replied to them in this rebuttal letter. All modifications introduced in the text can be visualized using the track changes tool of Microsoft Word.

1. Line numbering: why does it only start on page 7?

Reply: Thanks for spotting this error. We have now renumbered all lines starting from page 1. To facilitate the task of identifying where changes were introduced in the revised version of the manuscript, we also included the line number in the responses.

2. Check spelling in the entire document and correct typos. E. g: in the abstract it reads “We used PCR for the initial screening of samples for the presence of intestinal protest species”, when it should be “protist”.

Reply: Thanks for spotting these errors. The whole body of the main manuscript has been thoroughly revised, and typos found corrected.

3. Was there any sample size calculation? I would expect to have this explanation, particularly given that this was a prospective, transversal epidemiological study (cross-sectional?)

Reply: Please note that, because of the volunteering nature of the survey, we adopted a nonprobability convenience sampling strategy. This point has been now highlighted in current lines 188-189. To back up the statement, current reference [44] has been added into the Reference section.

4. How were participants for the study selected? Were there any specific inclusion and exclusion criteria? This is relevant, especially since risk factors are evaluated.

Reply: Reviewer #1 raised an important question. As commented in our answer to your comment #3, the study was based on a convenience sampling without specific inclusion/exclusion criteria. The potential bias associated with this sampling strategy has been acknowledged in the paragraph devoted to explaining the limitation of the survey in current lines 594-597 of the manuscript.

5. Authors should indicate the timeframe for the study, i. e. when was the sample collection and data collection conducted in Zambia?

Reply: Sampling was conducted between May-August 2023. This information has been now added in current line 188 of the manuscript.

6. The authors indicate that the samples were stored for some time in the University Teaching Hospital (Lusaka) for some time, but where were the samples finally processed? This is only specified unambiguously for *Blastocystis*-positive and *Blastocystis*-suspected samples which were further analysed at the US Department of Agriculture.

Reply: We thank Reviewer #1 for noticing this omission. Stool samples were shipped to the Parasitology Reference and Research Laboratory of the National Centre for Microbiology (Majadahonda, Spain) for DNA extraction and purification and PCR testing. *Blastocystis*-positive DNA samples requiring additional (NGS) analyses were subsequently shipped to USDA for this specific purpose. All this information has been clarified in lines 219-221 and 285-290 of the manuscript.

7. For statistical analysis of epidemiological data, the authors presented p-values in Table 2. What statistical tests were applied? I assume it was chi-square or Fisher’s exact test, if that is the case, was a method to correct for multiple testing (Bonferroni) applied when more that two categories in a variable was compared? Examples: age group (5-9 years, 10-14 years, 15-18 years and unknown); drinking water. And for those variables with the category Unknown, how was it treated? Is it included in the statistical test or is it excluded?

Reply: Following Reviewer #1´s advice we complemented our statistical analyses implementing Bonferroni correction for multiple comparisons. Consequently, the adjusted alpha level was set to 0.01667 instead of 0.05. Due to this adjustment, the variables “age group”, “presence of *G. duodenalis*” and “presence of *Cryptosporidium* spp.” lost their statistical significance in the reanalysed results. Samples for which information for a given variable was unknown were not included in the analysis. All this information has been now included in current lines 304-310 of the Material and Methods section and in the footnote of Table 2 in line 329. The *P*-values for variable age groups in *G. duodenalis* and *Cryptosporidium* spp. has been modified in Table 2. Accordingly, we have also eliminated from the Abstract and Results sections those results showing preliminary statistical significance unconfirmed by the Bonferroni correction.

8. In the “3.2. Analysis of risk factors potentially associated with infections by intestinal protists” sub-section, authors state that “*Giardia duodenalis* and *Cryptosporidium* infections were significantly more frequent in children belonging to the 10–14 years age group (*P*-values: 0.01 and 0.02, respectively)”, however, the data on Table 2, shows that the *Cryptosporidium* was more frequent in the 5-9 years age group, with 10 of the 11 infected participants. In addition, this would be in line with what has been documented by the GEMS study, where it was much more common in younger children (<2 years).

Reply: We thank Reviewer #1 for spotting this mistake. This information has been now corrected in current lines 348-353 of the manuscript.

9. The authors present the frequency of the protists according to different variables (very often referring to them as risk factors). Both in the Results and the Discussion they state that children with a certain specific characteristic “… were significantly more likely …” to be infected/carry a given protist. However, since they only presented a comparison of frequencies between groups and not the results of association analysis (using, for example, logistic regression or other methods) the wording should be revised carefully, as it can be misleading. I understand that association analysis might be beyond the scope of this work.

Reply: We agree with the concern raised by Reviewer #1. In the absence of more complex statistical analyses, we have avoided the use of the terms “risk factor” or “risk analysis” through the Results and Discussion sections. These terms have been replaced by most appropriate terms such as “variable” and “likelihood”, among others.

**Reviewer #2**

This study evaluated the asymptomatic intestinal protozoan infection in Zambia. High-accuracy molecular diagnostic approaches, including specific PCR and next-generation sequencing, were first utilized in this survey. The findings provided the prevalence of *Cryptosporidium* sp., *Giardia duodenalis*, *Entamoeba dispar*, and *Blastocystis* sp. in school-aged healthy individuals in Chongwe and Kafue districts in Lusaka Province. In addition, detailed information on subtypes and genetic variations was also documented. This investigation highlights the importance of asymptomatic carriers of intestinal protists in public health in the sub-Sahara area. Generally, the manuscript seems almost acceptable in the current version; however, some issues should be addressed before further processing.

Reply: We thank Reviewer #2 for his/her initial positive appraisal of manuscript PNTD-D-24-01392. We have considered all the comments and suggestions raised during the appraisal of the manuscript and thoroughly replied to them in this rebuttal letter. All modifications introduced in the text can be visualized using the track changes tool of Microsoft Word.

1. Lines 33 to 38: The samples were collected from Kafue and Chongwe districts in Lusaka Province, Zambia. The epidemiology investigation provides valuable information for African countries and regions with similar geographical characteristics. Therefore, a map containing the geographical, hydrological, and livestock-keeping facilities in Lusaka Province, especially the Kafue and Chongwe districts, is recommended to be provided.

Reply: A map showing the location of the sampled primary schools within the Chongwe and Kafue Districts in Lusaka province has been now added to the manuscript. Subsequent Figures have been renumbered consequently.

2. Page 6 to lines 13: It is appreciated that the authors organized a list to emphasize the previous epidemiological surveys and the utilities of relatively low-sensitivity methods in Zambia (Table 1). However, the table, if possible, should be introduced/ discussed more or moved as a supplementary file.

Reply: We feel that Table 1 is relevant in the main body of the manuscript as it provides the reader a clear summary at a glance of what is known about the epidemiology (in terms of prevalence and molecular diversity) of intestinal protists in humans and domestic animals in Zambia. We believe that this information is very useful to put the reader in the right epidemiological frame when interpreting the data generated in the present survey. The Table conveys three clear points: i) most conducted surveys used for detection conventional methods (e.g., microscopy, IFA, ELISA); ii) consequently, there is a clear lack of information on the molecular diversity of enteric protists, particularly for *G. duodenalis* and *Blastocystis* sp.; and iii) the vast majority of the surveys were geographically restricted to the Lusaka province, meaning that epidemiological scenario showed in Table 1 may differ in other Zambian geographical regions. Please note that many of these conclusions were already stated in current lines 163-167 of the manuscript. Following the advice of Reviewer #2, we have expanded this paragraph in current lines 165-167 of the manuscript.

3. Lines 169 to 173: The co-infection of individuals with two or more protists was mentioned. However, further analysis was expected, such as the origin of samples, the livestock contact, etc. The discussion section should also include the possible speculation of the co-infection with different protists.

Reply: Please note that origin (location) of infections were statistically analysed for single parasites. This analysis was not conducted for co-infected individuals because the limited number of cases would not reach statistical power. For the very same reason, the potential zoonotic origin of some of the infections has been discussed from the available molecular data generated in the survey, which in our opinion are more robust and appropriate to tackle this specific purpose. Please note that this information has already been included in current lines 527-538 in the case of *Cryptosporidium*. In the case of *Giardia*, and following the advice of Reviewer #2, we have added a sentence in current lines 516-517 of the Discussion section to comment that, based on the absence of animal-adapted assemblages C-F we believe that most of the *Giardia* infections detected in the present survey are expected to be of anthropogenic nature.

4. Lines 187 to 191: The authors mentioned that “all 79 faecal DNA samples with a positive result …”, but only 31 could be amplified and typed. The detailed detection strategy was stated in the method section (from line 70). However, for clarity and to make the audience easy to understand, the screening process needed to be introduced in the result section, too.

Reply: We believe that the pipeline followed for the detection and genotyping of *G. duodenalis* is appropriately described in current lines 229-238 of the Material and Methods section. To facilitate the access to this information we have added a call (see subsection 2.5) in current line 358 of the manuscript.

5. Lines 244 to 249: The authors stated that “A total of 97 *Blastocystis*-positive samples were successfully genotyped”, and the proportion of subtypes was revealed. It was unknown why the following paragraph started with “a subset of samples (n=45) …”. What were the missing samples out of the subset 45? Again, although the method section was stated, the origin of the subset should also be mentioned logically in the result section.

Reply: We thank Reviewer #2 for bringing to our attention this issue. The subset of 45 samples reanalysed by NGS included 40 *Blastocystis*-positive samples (from the 97 samples confirmed by Sanger sequencing) and an additional five *Blastocystis*-suspected samples (unconfirmed by Sanger sequencing). To avoid misleading interpretations, this information has been now clarified in current lines 285-290 of the Material and Methods section and in lines 416-418 of the Results section.

6. Lines 268 to 276: The whole paragraph about intra-subtype variants, coupled with Table 5, is not easy to understand.

Reply: This paragraph has been rewritten and reorganized to make information about intra-subtype variation more understandable (lines 438-451).

7. Table 5 showed the subtype, No. of unique ST variants, No. of samples containing variants, and GenBank accession number. Nonetheless, “… with 27 unique variants among 28 ST2-positive samples…”, “… with 19 unique variants among the 23 ST1-positive samples”, and “…with only six unique variants among 14 ST2-positive samples”: these “positive samples (28, 23, and 14 positive samples mentioned above)” could not be found in the table.

Reply: The number of samples positive for each ST is found in Table 4 while Table 5 highlights the number of unique sequence variants of each subtype, the number of samples in which each unique variant was observed, and the accession number for each unique sequence variant. Additional information was added to the text on lines 438-451.

8. Moreover, “…relatively evenly distributed variants among samples”, “with only three unique variants”, and “… detected in more than five children” (lines 272 to 273): The statements “three unique variants” and “more than five children” were not found Table 5.

Reply: This section has been edited for clarity (Lines 438-451).

9. Lines 274 to 276: “the multiple unique variants of ST1 and ST2…” and “multiple variants of ST3 were only detected in a single sample”. The same issue as above, the sample information was absent.

Reply: This information can be found in Table 4 which is hopefully now clearer in the text (Lines 438-451).

10. Most importantly, it was easy to imagine that NGS generated massive amounts of data, and the authors tried to explain all the valuable information in this manuscript. However, the authors did not mention the reason for discussing unique intra-subtype variants within samples.

Reply: As suggested, this consideration has been added to the discussion on lines 560-565.

11. Lines 317 to 323: the paragraph should be moved to line 365, where the *Blastocystis* was discussed.

Reply: Please note that the Discussion section follows a carefully chosen structure. First, we describe the presence (prevalence) of the investigated intestinal protists including *G. duodenalis* (current lines 465-472), *Cryptosporidium* spp. (current lines 473-482), *E. histolytica*/*dispar* (current lines 483-491) and *Blastocystis* sp. (current lines 492-498). Then, and following the same scheme, we started a section of the Discussion specifically devoted to comment the genotyping data generated in the present survey for *G. duodenalis* (current lines 499-517), *Cryptosporidium* spp. (current lines 518-540), and *Blastocystis* sp. (current lines 541-572). In order to maintain the flow and readability of the manuscript, we prefer therefore to keep the text in its current form.

12. The authors tried to elucidate sub-assemblages and subtypes of *G. duodenalis* and *Blastocystis* sp. as possible, respectively. Intriguingly, detection of the sub-assemblages of *G. duodenalis* at least 6 rounds of PCR were performed, whereas NGS was conducted to determine the subtypes of *Blastocystis* sp. It was noticed that the NGS was solely designed for *Blastocystis* sp. identification; why did the authors not conduct NGS to detect both protists, especially since they caused the most frequent co-infection in tested samples?

Reply: The NGS method used in this study is specific for *Blastocystis* and allows mixed ST detection. It has been validated and use for this purpose in many previous studies in animals and humans. For parasites, non-specific NGS strategies, even those that are based on 18S amplicons, lack the sensitivity of parasite specific assays and often miss parasites that can be easily detected with amplicon-based assays. That is why we used an amplicon-based assay for *Blastocystis* and not a non-parasite specific assay. With regards to *Giardia*, we are interested in testing our recently developed amplicon-based NGS assay for *Giardia* in human samples, however that was outside of the scope of this study.

13. The NGS-based subtype determination of *Blastocystis* sp. was performed in this study. It is known that the protist might not be pathogenic or even beneficial for human hosts. Was it worth the effort to determine the subtypes?

Reply: It is true that some recent metagenomic studies have proposed *Blastocystis* as potentially beneficial. However, there is a great deal we do not know about the role of *Blastocystis* in health and disease and how ST diversity plays into those outcomes in humans or other hosts. Several other recent studies draw the opposite conclusion about the role of *Blastocystis* in promoting health. NGS is a very powerful tool able not only to detect mixed infections/colonizations by different *Blastocystis* STs in the very same sample, but also to identify intra-subtype diversity. Taking advantage of this potential, our research group was able to differentiate genetic variants within *Blastocystis* ST10 (see Santín et al. J Eukaryot Microbiol. 2024;71:e12998. doi: 10.1111/jeu.12998), opening the door to questions such as if differences in pathogenicity and host specificity could be better explained at the intra-subtype level rather than at the subtype level. Current (still unpublished) evidence by our research team strongly suggests that certain *Blastocystis* intra-subtype genetic variants are specifically found in certain host species. Considering the relevance of these preliminary data, we feel that the hypothesis should be tested under any available epidemiological scenario. This is exactly the point to justify the effort conducted on *Blastocystis* using NGS-based subtype identification in the present survey.
